# The prognostic effect of perineural invasion in esophageal squamous cell carcinoma

**DOI:** 10.1186/1471-2407-14-313

**Published:** 2014-05-05

**Authors:** Jie-Wei Chen, Jing-Dun Xie, Yi-Hong Ling, Peng Li, Shu-Mei Yan, Shao-Yan Xi, Rong-Zhen Luo, Jing-Ping Yun, Dan Xie, Mu-Yan Cai

**Affiliations:** 1 Sun Yat-sen University Cancer Center; State Key Laboratory of Oncology in South China; Collaborative Innovation Center for Cancer Medicine, Guangzhou, China; 2 Department of Pathology, Sun Yat-sen University Cancer Center, No. 651, Dongfeng Road East, Guangzhou 510060, China; 3 Department of Anesthesiology, Sun Yat-sen University Cancer Center, Guangzhou, China

**Keywords:** Perineural invasion, Prognosis, Esophageal squamous cell carcinoma

## Abstract

**Background:**

Perineural invasion (PNI) is correlated with adverse survival in several malignancies, but its significance in esophageal squamous cell carcinoma (ESCC) remains to be clearly defined. The objective of this study was to determine the association between PNI status and clinical outcomes.

**Methods:**

We retrospectively evaluated the PNI of 433 patients with ESCC treated with surgery between 2000 and 2007 at a single academic center. The resulting data were analyzed using Spearman’s rank correlation, the Kaplan-Meier method, Cox proportional hazards regression modeling and Harrell’s concordance index (C-index).

**Results:**

PNI was identified in 209 of the 433 (47.7%) cases of ESCC. The correlation analysis demonstrated that PNI in ESCC was significantly correlated with tumor differentiation, infiltration depth, pN classification and stage (*P* < 0.05). The five-year overall survival rate was 0.570 for PNI-negative tumors versus 0.326 for PNI-positive tumors. Patients with PNI-negative tumors exhibited a 1.7-fold increase in five-year recurrence-free survival compared with patients with PNI-positive tumors (0.531 *v* 0.305, respectively; *P* < 0.0001). In the subset of patients with node-negative disease, PNI was evaluated as a prognostic predictor as well (*P* < 0.05). In the multivariate analysis, PNI was an independent prognostic factor for overall survival (*P* = 0.027). The C-index estimate for the combined model (PNI, gender and pN status) was a significant improvement on the C-index estimate of the clinicopathologic model alone (0.739 *v* 0.706, respectively).

**Conclusions:**

PNI can function as an independent prognostic factor of outcomes in ESCC patients, and the PNI status in primary ESCC specimens should be considered for therapy stratification.

## Background

Esophageal cancer is one of the most aggressive cancers worldwide, and its incidence rate has increased significantly in recent years [1,2]. As the dominant type of esophageal cancer in China, esophageal squamous cell carcinoma (ESCC) has a generally poor prognosis due to the lack of effective clinical methods for its early detection. Recently, improvements in diagnostic modalities and the development of a combination treatment of surgery, radiation and chemotherapy have improved the outcomes for this cancer [3]. Despite these advances, the prognosis in patients with ESCC remains poor, with an overall 5-year survival of 15-34% [4,5]. Given the poor prognosis of ESCC and its high incidence, it is increasingly important to understand the initiation and progression of ESCC and to identify the prognostic factors most associated with it.

An appropriate risk-stratified selection for adjuvant treatment trials is paramount, considering the high cost and toxic side effects of chemotherapeutic drugs. A variety of prognostic characteristics, including tumor location, size, differentiation, infiltration depth, lymph node involvement and distant metastasis, have been proposed as relevant factors for predicting the outcomes of patients with ESCC [6]. These characteristics have been incorporated into a proposed prognostic monogram designed to predict patients’ survival [7]. Although the currently proposed TNM system shows conformable prognostic accuracy, a demand remains for increasing the accuracy of the existing system for predicting outcomes.

Perineural invasion (PNI) involves cancer cells surrounding nerve fibers and entering the perineurium, spreading local infiltration and metastasis [8]. Cancer cells found in the perineurium are indicative of neural invasion [9]. PNI is regarded as a prominent characteristic of ESCC, as PNI is frequently observed in ESCC [10]. The PNI status has been found to be significantly correlated with poor survival in ESCC patients, as evidenced by univariate analysis [11]. However, Ochiai et al. reported that PNI was not a significant prognostic parameter in esophageal cancer [12]. Moreover, PNI in N0 esophageal cancer was found to have no association with cancer-specific survival following curative esophagectomy in a univariate statistical analysis [13]. Although PNI has been investigated in both esophageal SCC and adenocarcinoma, its incidence and prognostic value based on ESCC in high prevalence in a region, with a focus on PNI, has not been clarified. To address these issues, we employed a large cohort of patients with ESCC in a region in where it has a high prevalence to determine the association between PNI status and clinical outcomes.

## Methods

### Patients

A total of 433 patients with ESCC who underwent curative esophagectomies between October 2000 and May 2007 were randomly selected from the Department of Pathology of Sun Yat-sen University Cancer Center (Guangzhou, China). The selective criteria included (1) no adjuvant treatment before the surgery; (2) a complete resection of the tumor; (3) a negative incised margin; (4) no distant metastasis; and (5) detailed and complete follow-up data.

We collected clinicopathologic data that included the patient’s age, gender, tumor location, tumor size, differentiation, TNM stage, infiltration depth, lymph node status, vascular invasion and recurrence. These data are detailed in Table 1. Tumor differentiation was determined based on the criteria proposed by the WHO’s classification of Tumours of the Digestive System (2010 version). The tumor stage was defined according to the American Joint Committee on Cancer/International Union Against Cancer TNM (tumor-node-metastasis) classification system (2010 version). The patients were followed every 3 months for the first year and then every 6 months for the next 2 years and then annually after surgery. Tumor recurrences (including local recurrence or metastasis) were detected by ultrasonography, CT or MRI. The time of the detection of recurrence was unknown until the patient died from ESCC, and the time to death was used instead. The Institute Research Medical Ethics Committee of Sun Yat-sen University Cancer Center granted approval for this study.

**Table 1:** Correlation of perineural invasion with patients’ clinicopathological features in primary esophageal squamous cell carcinomas

**Variables**	**Perineural invasion**			
	**Cases**	**Absent**	**Present**	***P*** value*
Age (years)				0.083
≤ 57.0^†^	230	110 (47.8%)	120 (52.2%)	
> 57.0	203	114 (56.2%)	89 (43.8%)	
Gender				0.502
Female	112	61 (54.5%)	51 (45.5%)	
Male	321	163 (50.8%)	158 (49.2%)	
Location				0.098
Upper	28	14 (50.0%)	14 (50.0%)	
Middle	298	145 (48.7%)	153 (51.3%)	
Lower	107	65 (60.7%)	42 (39.3%)	
Tumor size (cm)				0.952
≤ 4^‡^	277	143 (51.6%)	134 (48.4%)	
> 4	156	81 (51.9%)	75 (48.1%)	
Differentiation				0.017
Well	67	41 (61.2%)	26 (38.8%)	
Moderate	288	153 (53.1%)	135 (46.9%)	
Poor	78	30 (38.5%)	48 (61.5%)	
pT status				0.036
T1	20	15 (75.0%)	5 (25.0%)	
T2	104	59 (56.7%)	45 (43.3%)	
T3	309	150 (48.5%)	159 (51.5%)	
pN status				< 0.0001
N0	233	137 (58.8%)	96 (41.2%)	
N1	111	59 (53.2%)	52 (46.8%)	
N2	71	25 (35.2%)	46 (64.8%)	
N3	18	3 (16.7%)	15 (83.3%)	
Stage				< 0.0001
I	25	19 (76.0%)	6 (24.0%)	
II	238	138 (58.0%)	100 (42.0%)	
III	170	67 (39.4%)	103 (60.6%)	

*Chi-square test; ^†^Median age; ^‡^Median size.

### Pathological evaluation

The patients’ records and original histopathologic slides were independently reviewed by 2 pathologists with expertise in gastrointestinal pathology (S.-Y. Xi and M.-Y. Cai) who were blinded to the pathological diagnoses and outcome data. Discrepancies were resolved by the simultaneous re-examination of the slides by both pathologists with a double-headed microscope. The presence of PNI was carefully evaluated on hematoxylin and eosin (H&E)-stained slides. The cases were examined for the presence or absence of PNI, which was defined as the presence of viable tumor cells in the perineural space [14]. Descriptions of PNI have included cancer cells within any layer of the peripheral nerve sheath, from the abutment of cancer cells with the perineurium to small clusters of cancer cells within the nerves [15]. When tumor cells are not located inside of the nerve sheath but are in close proximity to the nerve in the perineural environment, at least 33% of the circumference of the nerve should be surrounded by tumor cells to diagnose PNI; anything less than 33% represents focal abutment and not invasion [15].

### Statistical analysis

The correlation between PNI and the clinicopathologic variables of the ESCC patients was evaluated by a χ2-test. For the univariate analysis, survival curves were obtained with the Kaplan-Meier method, and the differences between the groups in survival were determined by the log-rank test. Multivariate survival analyses were performed with the Cox proportional hazard regression model. Harrell’s concordance index (C-index) was employed to assess the model’s prognostic accuracy in the multivariate analysis. A *P* value from a two-tailed test that was less than 0.05 was considered to be statistically significant. The statistical analysis was performed with the SPSS statistical software package (SPSS Standard version 13.0; SPSS, Chicago, IL, USA) and R, version 3.0.1 (http://www.r-project.org/).

## Results

### Patient characteristics

The characteristics and pathological features of our ESCC cohort’s patients are detailed in Table 1. The cohort included 321 (74.1%) males and 112 (25.9%) females, with a mean age at the time of resection of 57.2 years (range, 30–81). The median follow-up of all patients was 39.0 months, ranging from 1.0 months to 115.0 months. Sixty-nine percent of the tumors (298 of 433) occurred in the middle segment of the chest, and the remaining tumors occurred in the upper (6.5%) and lower (24.7%) segments of the chest.

### Relationship between PNI and clinicopathologic features in ESCC

**Figure 1:**
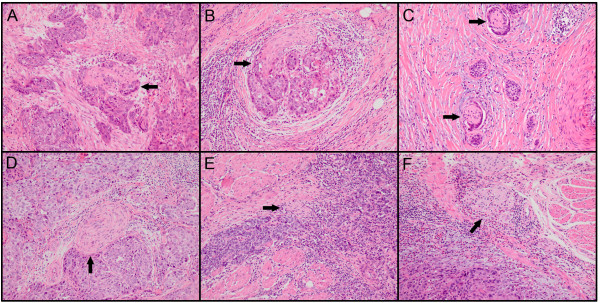
**The status of perineural invasion (PNI) in ESCC specimens.** Tumor cells located within any of the endoneurium **(A)**, perineurium **(B)** and epineurium **(C)** of the peripheral nerve sheath are clear examples of PNI. When tumor cells are not located inside of the nerve sheath but are in close proximity to the nerve in the perineural environment, at least 33% of the circumference of the nerve should be surrounded by tumor cells to diagnose PNI **(D)**; anything less than 33% represents focal abutment and not invasion **(E)**. When tumor cells are not in close proximity to the nerve in the perineural environment **(F)**, it represents negative finding (hematoxylin-eosin staining, ×100).

PNI was defined as tumor cells within any layer of the peripheral nerve sheath or tumor cells in the perineural space that involved at least 33% of the nerve circumference. The patterns of PNI in ESCC specimens were shown in Figure 1. PNI was identified in 209 (47.7%) of the 433 patients. A further correlation analysis demonstrated that the presence of PNI was significantly correlated with tumor differentiation, infiltration depth, pN classification and ESCC stage (*P* < 0.05, Table 1). There was no significant association between the presence of PNI and other clinicopathologic features, such as the patient’s age, gender and location and size of the tumor (*P* > 0.05, Table 1).

### Role of PNI as prognostic factor of outcome in ESCC patients

**Figure 2:**
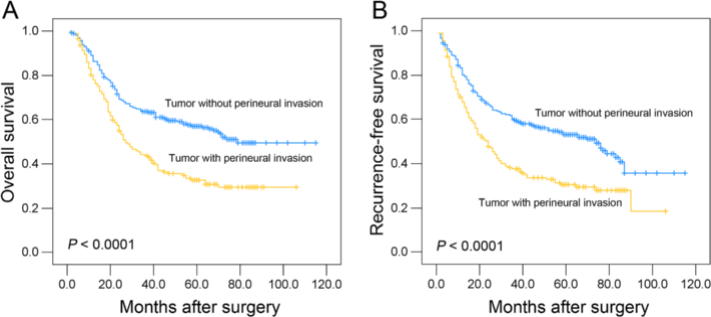
**The association between perineural invasion and ESCC patients’ survival (log-rank test).** Kaplan-Meier survival analysis of perineural invasion for overall survival **(A)** and recurrence-free survival **(B)** in ESCC patients.

The prognostic significance of PNI as well as other clinicopathologic variables was investigated by univariate analyses. The PNI status (*P* < 0.0001), patient gender (*P* = 0.016), tumor differentiation (*P* = 0.021), pT status (*P* = 0.002), pN status (*P* < 0.0001) and tumor stage (*P* < 0.0001) influenced the patients’ overall survival (Table 2). Patient age and the size and location of the tumor did not significantly affect outcomes. The 5-year overall survival rate was greater for patients with PNI-negative tumors than for patients with PNI-positive tumors (0.570 *v* 0.326, respectively; *P* < 0.0001; Figure 2A). Similar results were found for recurrence-free survival. Patients with PNI-negative tumors exhibited a 1.7-fold increase in five-year recurrence-free survival compared with patients with PNI-positive tumors (0.531 *v* 0.305, respectively; *P* < 0.0001; Figure 2B).

**Table 2:** Univariate analysis of perineural invasion and clinicopathologic variables in patients with primary esophageal squamous cell carcinoma (log-rank test)

**Variables**	**Cases**	**Mean survival (months)**	**Median survival (months)**	***P*** **value**
Age (years)				0.657
≤ 57.0^†^	230	62.5	42.0	
> 57.0	203	60.1	58.0	
Gender				0.016
Female	112	72.6	NR	
Male	321	57.7	41.0	
Location				0.083
Upper	28	68.5	79.0	
Middle	298	56.2	41.0	
Lower	107	70.7	63.0	
Tumor size (cm)				0.076
≤ 4^‡^	277	66.1	63.0	
> 4	156	54.3	38.0	
Differentiation				0.021
Well	67	61.9	64.0	
Moderate	288	65.5	64.0	
Poor	78	44.4	26.0	
pT status				0.002
T1	20	58.2	NR	
T2	104	71.2	74.0	
T3	309	57.9	38.0	
pN status				< 0.0001
N0	233	79.3	NR	
N1	111	48.2	40.0	
N2	71	27.4	16.0	
N3	18	19.1	11.0	
Stage				< 0.0001
I	25	67.6	NR	
II	238	76.4	NR	
III	170	34.9	22.0	
Perineural invasion				< 0.0001
Absent	224	71.4	79.0	
Present	209	47.7	27.0	

^†^Median age; ^‡^Median size; *NR* indicates not reached.

**Figure 3:**
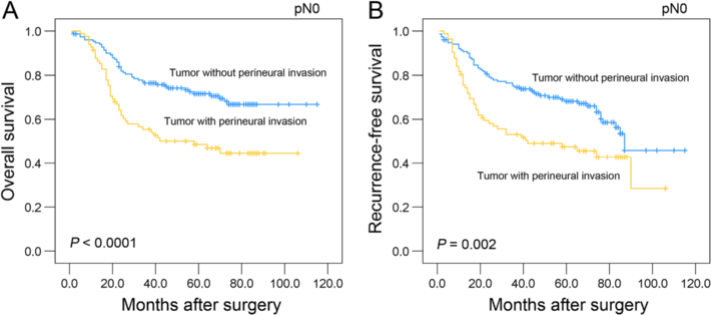
**Univariate survival analysis with regard to perineural invasion in the subset of ESCC patients with node-negative disease.** The presence of perineural invasion was identified as a prognostic predictor of overall survival **(A)** and recurrence-free survival **(B)** in ESCC patients with node-negative disease.

Among the node-negative patients (233 of 433 patients, 53.8%), PNI remained a significant predictor of poor outcomes. The 5-year overall survival rate was more than 1.4-fold greater among patients with PNI-negative tumors than among patients with PNI-positive tumors (0.716 *v* 0.486, respectively; *P* < 0.0001; Figure 3A). The recurrence-free survival at 5 years was 0.682 in patients with PNI-negative tumors compared with 0.475 in patients with PNI-positive tumors (*P* = 0.002; Figure 3B).

### PNI status is an independent prognostic factor of outcome in ESCC patients

A Cox multiple regression model was utilized to assess the influence of all significant covariates on overall survival in ESCC patients who underwent curative resections. After controlling for patient gender, tumor differentiation, pT status, pN status and stage, the multivariate analysis confirmed that the presence of PNI was a significant independent prognostic factor for poor overall survival (hazard ratio, 1.374; 95% CI, 1.037-1.820; *P* = 0.027; Table 3). Of the other parameters, patient gender and pN status were evaluated as independent prognostic factors for the overall survival of ESCC patients (*P* < 0.05; Table 3). Applying Harrell’s C-index to test the predictive ability of integrating PNI into the prognostic model in ESCC patients, our results showed that the PNI status improved the predictive ability when compared with the clinicopathologic model (C-indexes of 0.739 *v* 0.706, respectively).

**Table 3:** Cox multivariate analyses of prognostic factors for overall survival

**Variables**	**Hazard ratio**	**95%CI**	***P*** **value**
Gender (female *v* male)	1.379	1.005-1.891	0.046
Differentiation (well *v* moderate *v* poor)	1.116	0.897-1.389	0.324
pT status ( T1 *v* T2 *v* T3)	1.146	0.831-1.579	0.406
pN status (N0 *v* N1 *v* N2 *v* N3)	1.687	1.339-2.125	< 0.0001
Stage (I *v* II *v* III)	1.195	0.773-1.848	0.422
Perineural invasion (absent *v* present)	1.374	1.037-1.820	0.027

CI, confidence interval.

## Discussion

PNI is the process of neoplastic invasion of the nerves, and it is also an important pathway by which tumor’s progress and spread to the adjacent tissues or organs. It is commonly detected in human cancers of the pancreas and biliary tract [16-18]. It has been found to be a crucial route for the local spread of tumors that are associated with poor outcomes in various types of human cancers. However, the role of PNI status in ESCC and its utility to clinicians continue to be debated. In our study, we assessed a retrospective collection of data to determine the prognostic value of PNI for the survival of patients with ESCC who underwent curative esophagectomies. Moreover, our particular interest is in determining the potential effects of PNI in node-negative ESCC patients.

Our results demonstrated that the presence of PNI was observed in 47.7% of patients with ESCC as evaluated on H&E-stained slides. However, less than 30% of the PNI-positive tumors were detected in esophageal cancer (including SCC and adenocarcinoma) in previous studies [10,12]. Differences in the histological types and PNI definitions may contribute to this discrepancy. In the current study, we utilized a broader definition inclusive of both of the circumstances: cancer cells surrounding at least one-third of the nerve without invading through the nerve sheath, as well as tumor cells within any of the 3 layers of the nerve sheath [19-22]. Furthermore, the increased detection rate observed in our study is most likely because the pathologic analysis was performed by pathologists with expertise in PNI who were blinded to the follow-up data in this study. Consistent with our study, PNI has been recognized in many series as a prevalent pathological characteristic of gastric cancer and is reported in up to 73% of this tumor at the time of resection [23]. PNI rates were much higher in pancreatic cancer (70-100%) and in biliary tract cancer (75-85%) [16,24]. Our findings suggest that PNI is a common pathologic feature of ESCCs removed by radical esophagectomy.

A further correlation analysis revealed that the presence of PNI in ESCCs was significantly associated with tumor differentiation, infiltrate depth, pN status and stage. Studies in other cancers have led to similar conclusions. For instance, Ochiai et al. reported that the presence of PNI in esophageal cancer was closely correlated with the depth of invasion [12]. Liebig et al. found that PNI was associated with a more advanced stage and poor tumor differentiation in colorectal cancer [20]. Previous studies also showed that evidence of PNI on a prostate needle biopsy is predictive of a higher T stage and extracapsular invasion at resection [25,26]. Furthermore, the presence of PNI was found to be correlated with tumor size, margin status, lymph node metastasis and AJCC stage in patients with pancreatic ductal adenocarcinoma [14]. Taken together, these data suggest that PNI plays an important role in the development and/or progression of human cancers.

Our findings of the PNI status and its correlation with ESCC patients’ outcomes are consistent with the results of other groups. In 1999, Torres et al. showed a significant association between the presence of PNI and the poor survival of patients with ESCC as evidenced by a univariate analysis [11]. Other groups have reported similar results [27-29], in which PNI was a significant pathologic parameter in head and neck cancers, heralding decreased survival, increased locoregional recurrence rates and a shorter time to relapse. In a more recent study, Liegig et al. suggested that PNI is correlated with decreased survival on a multivariate analysis and established that PNI is an independent predictor of outcomes in colorectal cancer patients [20]. Importantly, PNI in pT3N0 rectal cancer is also found to be an independent risk predictor of a local recurrence [30]. Notably, other published reports show no significant prognostic value for PNI to predict the outcomes in patients with esophageal squamous cell carcinoma and adenocarcinoma [12,13]. Taken together, differences in the clinicopathologic characteristics among cohorts, geographic backgrounds, definition of PNI, patient heterogeneity, small sample sizes and different definitions of end points (disease-free, cancer specific or overall survival) may contribute to the controversial results. In our study, the findings of a significant association between the PNI status and clinical outcome in esophageal cancer may be strengthened by the single histological type and large sample size.

Generally, our results support the idea that the presence of PNI may improve the ability to discriminate among ESCC patients’ outcomes, especially for patients who are node-negative. It is well known that the pTNM stage and tumor differentiation are the best-established risk predictors for important aspects affecting the outcomes of patients with ESCC. These two variables, based on specific clinicopathologic features and the extent of the disease, may have reached their limits in providing critical information influencing patient prognosis and treatment strategies at the pN0 stage. Moreover, the outcomes of patients with the same stages following surgery are substantially different, and such large discrepancies have not been well understood [31,32]. Thus, there is a need for new objective strategies that can more effectively distinguish between patients with favorable and unfavorable outcomes. In the present study, our data support the concept that PNI, as detected by H&E staining, can identify ESCC patients with or without an aggressive clinical course and/or poor outcome at the pN0 stage. Thus, the evaluation of PNI may become a factor for predicting the prognosis and rendering a more tailored treatment strategy in patients with ESCC.

The retrospective nature of this study may be considered its major limitation, and it may have influenced these results. However, the study was strengthened by the fact that all of the histopathological slides were re-evaluated by the same gastrointestinal pathologists. Although our findings should be confirmed by prospective studies, we believe that our results contribute to the literature because it includes only patients with ESCC. To our knowledge, this is the first report to investigate the prognostic ability of PNI in an area of high ESCC prevalence; however, further external validation of our findings using pooled multicenter data is needed.

## Conclusions

In conclusion, the presence of PNI was a strong and independent predictor of a poor outcome, as indicated by the univariate and multivariate analyses. Adding the PNI model could improve the ability to discriminate ESCC patients’ outcomes. Our data suggest that PNI could function as an independent prognostic factor of outcomes in ESCC and support the consideration of the PNI status in primary ESCC specimens for therapy stratification. In particular, we advocate for the consideration of node-negative ESCC patients who are PNI positive for treatment with the effective adjuvant therapies that are currently available.

## Abbreviations

ESCC: Esophageal squamous cell carcinoma; PNI: Perineural invasion; SCC: Squamous cell carcinoma; TNM: Tumor-node-metastasis; H&E: Hematoxylin and eosin; C-index: Concordance index.

## Competing interests

The authors declare that they have no competing interests.

## Authors’ contributions

MYC is responsible for the study design. JWC and JDX performed the experiments and draft the manuscript. YHL, PL, SMY, SYX, JPY and DX participated in the data analysis and interpretation. All authors read and approved the final manuscript.

## Pre-publication history

The pre-publication history for this paper can be accessed here:

http://www.biomedcentral.com/1471-2407/14/313/prepub
